# The TRPV1 Channel Modulator Imidazo[1,2-a]Indole Derivative Exhibits Pronounced and Versatile Anti-Inflammatory Activity In Vivo

**DOI:** 10.3390/biomedicines14010060

**Published:** 2025-12-26

**Authors:** Pavel A. Galenko-Yaroshevsky, Anait V. Zelenskaya, Konstantin F. Suzdalev, Tatyana N. Popova, Aleksandra N. Kvetkina, Margarita M. Shamatova, Elena N. Chuyan, Marina Yu. Ravaeva, Roman A. Murashko, Tereza R. Glechyan, Alina V. Sergeeva, Narek N. Ishkhanyan, Olga N. Gulevskaya, Vladislav I. Chubinskiy-Nadezhdin, Evgenii D. Kryl’skii, Nadezhda A. Priymenko, Anna A. Klimovich, Elena V. Leychenko, Sergey A. Kozlov

**Affiliations:** 1 Department of Pharmacology, Kuban State Medical University, Krasnodar 350063, Russia; anait_06@mail.ru (A.V.Z.); t_g91@mail.ru (T.R.G.); ,; 2 Faculty of Chemistry, Southern Federal University, Rostov-on-Don 344090, Russia; 3 Department of Medical Biochemistry, Molecular and Cell Biology, Voronezh State University, Voronezh 394018, Russia; 4 Laboratory of Molecular Pharmacology and Biomedicine, Elyakov Pacific Institute of Bioorganic Chemistry, Far Eastern Branch, Russian Academy of Sciences, Vladivostok 690022, Russia; kvetkinaan@gmail.com (A.N.K.);; 5 Laboratory of Neuroreceptors and Neuromodulators, Shemyakin-Ovchinnikov Institute of Bioorganic Chemistry, Russian Academy of Sciences, Moscow 119997, Russia; 6 Institute of Biochemical Technologies, Ecology, and Pharmacy, V.I. Vernadsky Crimean Federal University, Simferopol 295001, Russia; elena-chuyan@rambler.ru (E.N.C.);; 7 Department of Public Health and Healthcare, Kuban State Medical University, Krasnodar 350063, Russia; 8 Department of Adaptive Physical Education, Kuban State University of Physical Education, Sports, and Tourism, Krasnodar 350015, Russia; 9 Group of Ionic Mechanisms of Cell Signaling, Department of Intracellular Signaling and Transport, Institute of Cytology, Russian Academy of Sciences, St. Petersburg 194064, Russia

**Keywords:** anti-inflammatory action, imidazo[1,2-a]indole derivative, NSAIDs, cytokines, TRPV1 inhibitor, animal models

## Abstract

**Background**: Recently, data have been published about the inhibitory effect at low nanomolar concentrations on the TRPV1 ion channel for a new indole derivative named SV-1010. This molecule has also been shown to have a strong analgesic effect in mice and rats. Since the biological target of SV-1010 is the TRPV1 ion channel, which plays an active role in inflammation, we conducted a series of animal tests to evaluate its potential as an anti-inflammatory agent. **Methods**: Nine different inflammatory agents were used to assess acute inflammation, and diclofenac was chosen as a positive control. Additionally SV-1010 effects in chronic proliferative and immunogenic inflammation models were also measured. **Results**: SV-1010 demonstrated a significant effect in most inflammatory tests, often surpassing that of diclofenac, and showed comparable efficacy to several other recognized anti-inflammatory drugs under certain conditions. The level of pro-inflammatory cytokines, TNF-α, IL-1β, and IL-6, exceeded after LPS administration was normalized to the non-LPS control group level by a dose of 0.1 mg/kg of SV-1010, and the effect was comparable to that of diclofenac at a dose of 12.5 mg/kg. The estimation by qPCR of the content of two enzymes, COX-2 and iNOS, which were increased by 10.8- and 19.4-fold, respectively, after LPS induction showed different molecular targets being utilized, manifested in the normalization of COX-2 content only after diclofenac treatment, and iNOS content only after SV-1010 treatment. **Conclusions**: Due to the simplicity of synthesis and low effective dose for mammal treatment, this compound can be interesting for a practice.

## 1. Introduction

Inflammation is the body’s natural response to various types of tissue damage, including infection by microbes, viruses, or fungi, as well as mechanical, thermal, or chemical injuries. It can also occur as an autoimmune reaction. Various mediators and modulators, such as histamine, serotonin, lysosomal enzymes, prostaglandins, and cytokines, are involved in the formation of acute inflammation, which is the first stage of the process. These substances not only reflect the type and severity of the initial damage but also determine how long and how quickly inflammation lasts, and whether it progresses to the next stage [1]. Thus, tissue inflammation and its resolving are heterogeneous processes that can be modeled in various ways in animals.

Epithelial cells in damaged tissue initiate the enzymatic breakdown of phospholipids and the production of arachidonic acid (AA). Upon release, AA generates numerous metabolites through the action of cyclooxygenases, lipoxygenases, and cytochrome P450. These metabolites are considered inflammatory bioactive lipids or eicosanoids [2].

However, some lipoxygenase products, such as lipoxins and prostacyclins, also exhibitanti-inflammatory activity. AA itself directly regulates inflammatory responses by modulatingthe activity of Toll-like receptor 4 (TLR4), preventing saturated fatty acid-induced activation of the pro-inflammatory TLR4 signaling pathway [3].

Thus, the AA-based inflammation model allows us to evaluate the effects of slow-developing inflammation, similar to allergic inflammation. A well-known eicosanoid, prostaglandin E2 (PGE2), which is derived from AA and acts as the main mediator of inflammation, has a dual effect. It can exert both pro-inflammatory and anti-inflammatory effects, depending on the receptors it binds to [4].

Carrageenan, a polysaccharide extracted from red algae, has been shown to cause histopathological changes in the paws of animals in a model of carrageenan-induced inflammation. The results demonstrate that carrageenan causes early phase expression of cyclooxygenase-2 (COX-2) gene in the central nervous system within one hour, leading to an increase in prostaglandin production in the following hours [5]. Additionally, immune cells that are involved in the inflammatory process contain a pre-synthesized amount of mediators such as histamine and serotonin, which can be quickly released, causing a rapid inflammatory response. Therefore, inflammation caused by endogenous amines reflects a rapid onset of inflammation.

Kinins are potent vasoactive peptides that are believed to be responsible for the main features of inflammation, as well as a number of other biological activities [6]. Aluminosilicate mineral kaolin can be used to assess the role of kinins in the pathogenesis of inflammation, as its phlogogenic action is associated with increased kinin production [7].

Bacterial endotoxins, such as lipopolysaccharides (LPS), are often used as inducers that can cause both acute exudative and systemic inflammation (SI). LPS binds to TLRs on the surface of immune cells, triggering a rapid response. Depending on the type of receptor and signaling pathway, LPS can induce the synthesis of pro-inflammatory cytokines or activate genes that regulate the cellular inflammatory response [8].

Zymosan is a mild leukopoiesis-stimulating agent that is derived from the yeast *Saccharomyces cerevisiae*. A recent study in a mouse model of colorectal hypersensitivity revealed increased TRPV1 channel activity compared to control mice not treated with zymosan [9]. The use of this model in the present study was extremely interesting, since the substance under study, an imidazo[1,2-a]indole derivative, inhibits currents through the TRPV1 ion channel heterologicaly expressed in the CHO cell line [10]. This molecule also exerts an analgesic effect at the supraspinal, spinal, and peripheral levels of pain sensitivity [10].

In this study, we investigated the anti-inflammatory effects of a single molecule using several models of acute exudative inflammatory conditions and compared these findings with those obtained from reference drugs. As per oral administration via the gastrointestinal tract, this molecule can be rapidly absorbed and exerts a sustained anti-inflammatory effect for several hours at ultra-low doses of 0.001–0.01 mg/kg. The study was also supported by a classic investigation of chronic inflammation and an arthritic model in which the molecule was also successful.

The side effects of NSAIDs associated with impaired synthesis of important eicosanoids have long been under control and are being carefully studied. Extensive modification of drugs in this group is unlikely to solve the problem, while switching to new molecular targets may help. The paper attempts to demonstrate the positive effect of the TRPV1 inhibitor on rodent inflammatory models in order to promote a new way of treating inflammation using this target in general.

## 2. Materials and Methods

### 2.1. Chemicals

The pure chemical substanceof 4-methoxy-N-((1-(4-methoxyphenyl)-1H-imidazo[1,2-a]-indol-9-yl)methylene)benzenemamonium chloride (named as SV-1010) was synthesized at the Southern Federal University, Rostov-on-Don, Russia, as described in [11]. The reference moleculesdiclofenac (chemical substance of series 23390D017, Armavir Biological Factory, Armavir, Russia), indomethacin (tablets, Ozon LLC, Zhigulievsk, Russia), nimesulide (tablets, Moscow, Ozon LLC, Russia), montelukast (tablets, Vertex FC, Saint-Petersburg, Russia), zileuton (chemical substance, Macklin, Shanghai, China), pelubio (tablets containing pelubiprofen, Akrikhin JSC, Staraya Kupavna, Russia), and celecoxib (tablets, Pharmproekt JSC, Saint-Petersburg, Russia) were used as positive controls.

### 2.2. Animals

Experiments were conducted on 40 male CD-1 mice weighing 28–32 g and 853 male Wistar rats weighing 210–320 g, maintained under standard vivarium conditions on a standard diet under the European Commission’s legislation (Directives 86/609/EEC, 2010/63/EU). Animal studies conform fully to the World Health Organization’s International Guiding Principles for Biomedical Research Involving Animals and are reported in compliance with the ARRIVE guidelines [12] and the Committee on Ethics of Laboratory Animal Handling Kuban State Medical University No. 119, 13 April 2023 (KSMU MH RF), of Elyakov Pacific Institute of Bioorganic Chemistry No. 01/25, 20 February 2025 (PIBOC FEB RAS) of Voronezh State University No. 42-02., 4 October 2021 protocols.

### 2.3. Experimental Research In Vivo Models

To investigate the effects of SV-1010 on various models of inflammation, animals were randomly assigned to experimental groups of 8–15 individuals each.

#### 2.3.1. Acute Exudative Inflammation

Acute exudative inflammation was induced in rats by subplantar injection of various inflammatory agents according to the protocols [13]. The inflammatory agents used were: 1% carrageenan (i-carrageenan, Type V, Sigma-Aldrich, St. Louis, MO, USA); 10% kaolin (Bathsheba LLC, Novorossiysk, Russia); 0.005% PGE2 (Sigma-Aldrich, USA); 0.1% histamine (Sigma-Aldrich, USA); 0.01% serotonin (5-HT, Acros Organics, Geel, Belgium); 0.1% AA (Sigma-Aldrich, USA); 2% zymosan (Sigma-Aldrich); 0.1% bradykinin (EMD Milipore Corp., Billerica, MA, USA); and 100 μg/0.1 mL LPS of *Escherichia coli* (O111:B4, EMD Milipore, USA).

The paw volume was measured using a Life Science plethysmometerfrom IITC (USA). For carrageenan, the measurements were taken one and three hours after injection. For kaolin, the measurements were taken at one, two and twenty-four hours after injection, while for lipopolysaccharide, the measurements were taken two, four, six, seven and twenty-four hours after injection; for other inflammatory agents, the measurements were taken hourly for four hours.

SV-1010 was administered at doses of 0.001, 0.01, 0.1, 0.5, and 1 mg/kg intragastrically (i.g.), in a 1% potato starch solution, on an empty stomach (after 12 h of food deprivation), 1 h prior to administration of the inflammatory agent. Control animals received a 1% potato starch solution equivalent to that used for the test substances. Reference drugs were also administered to animals via i.g. at various experimental doses taken from literary sources or calculated via a transfer coefficients between species. Indomethacin was administered at doses of 5, 10, 15, and 20 mg/kg; diclofenac was administered at 2.5, 5, 7.5, 10, and 15 mg/kg; pelubiprofen was administered at a dose of 3 mg/kg, corresponding to a therapeutic human dose of 30 mg/kg, when converted to rat using the interspecies dose transfer formula; montelukast was administered in doses of 0.22, 0.44, and 0.88 mg/kg corresponding to human doses of 2.5, 5, and 10 mg/kg; and zileuton was administered at a dose of 60 mg/kg corresponding to a human dose of 600 mg/kg. Celecoxib was administered at a dose of 30 mg/kg.

The potency of the anti-inflammatory activity of SV-1010, as well as that of the reference drugs, was evaluated by the inhibitory activity (I, in %), which was determined using Equation (1):

I = (avrΔVc − avrΔV)/avrΔVc × 100%(1)

where avrΔVc or avrΔV is the paw volume average increase in the control or experimental group, respectively.

The average effective dose (ED_50_), which reflects the anti-inflammatory activity of a drug, was determined graphically. The therapeutic index (TI), which indicates the safety margin of a drug, was calculated by dividing the LD_50_ (the dose that causes 50% mortality in animals) by the ED_50_. The LD_50_ value of SV-1010 had been determined previously [10].

#### 2.3.2. Acute Systemic Inflammation

Acute SI was induced in mice by intraperitoneal injection (i.p.) of 0.1 mg/kg of LPS (*E. coli* 055:B5, from Sigma, USA). The animals were then administered an aqueous solution of SV-1010 and diclofenac at doses of 0.1 and 12.5 mg/kg, respectively, i.g., in a volume of 0.2 mL, one hour before the induction of inflammation. The negative control group received an equal volume of water for injection. After one and a half hours, blood was collected from all animals for hematological and immunological analysis. A complete blood count was performed using a Mindray BC-5000 veterinary hematology analyzer (China), and cytokine production was assessed through gene expression analysis using quantitative PCR.

#### 2.3.3. Chronic Proliferative Inflammation

Chronic proliferative inflammation was induced in rats by implanting a sterile 15 mg cotton ball subcutaneously on the back, using the method described by Zatsepina E.E. (2022) [14]. The rats were anesthetized with 400 mg/kg chloral hydrate before the procedure. The experimental drugs, SV-1010, diclofenac, indomethacin, nimesulide, and montelukast, were administered i.g. in a 1% potato starch solution, 1 h prior to cotton ball implantation. The drugs were administered daily for 7 days, beginning on the day of cotton ball implantation. On the eighth day of the experiment, the cotton ball and any granuloma that may have formed around it were removed and weighed. The “cotton wool granuloma” was then dried at 60 °C in a dry air incubator until it reached a constant weight. Exudative activity was measured by the difference in weight between the wet and dry granuloma. Proliferative activity was calculated by subtracting the weight of the original cotton wool ball from the weight of the dried granuloma.

#### 2.3.4. Chronic Immune Inflammation

Chronic immune inflammation, similar to adjuvant-induced or rheumatoid arthritis (RA), was simulated by i.p. injection of 0.1 mL of complete Freund’s adjuvant (CFA) (InvivoGen, Toulouse, France) into the right hind paw of rats. The anti-inflammatory, prophylactic, and therapeutic effects of SV-1010 were evaluated. For the prophylactic regimen, SV-1010 at doses of 0.01 and 0.1 mg/kg was administered i.g. in a 1% potato starch solution under fasting conditions via gavage daily for 14 days, beginning 1 day before CFA injection. The primary reaction (swelling of the right paw) was assessed using an IITC Life Science plethysmometer (USA) on days 3, 5, 7, 9, 11, 13 and 14 after the injection of CFA, and the secondary immunological reaction (swelling of the left paw) on the 14th day after the administration of CFA.

In the treatment regimen, SV-1010 at a dose of 0.01 mg/kg was administered i.g. daily for 12 days under fasting conditions, beginning 14 days after adjuvant injection. The primary (edema in the right paw) and secondary immunological (edema in the left paw) responses were assessed using a plethysmometer on days 14, 17, 20, 23, and 25 after adjuvant injection.

The anti-inflammatory effect of SV-1010 was evaluated by its inhibitory effect on paw edema growth, calculated using Equation (1). SV-1010 at a 0.01 mg/kg dose and diclofenac at a 12.5 mg/kg dose were administered i.g. every 24 h for 7 days starting on day 14 of RA onset. On day 21 after the experiment began, blood and heart tissue samples were collected for biochemical analysis.

### 2.4. Biochemical Analysis

The erythrocyte sedimentation rate (ESR) of rat blood was determined as described by Lugovskaya S.A (2006) [15]. The level of circulating immune complexes (CIC) in the blood serum was measured by precipitation with PEG 6000, followed by optical density measurement at 450 nm using a Shimadzu UV-1900i spectrophotometer (Shimadzu, Kyoto, Japan). The activity of creatine kinase MB (CK-MB) and aspartate aminotransferase (AST) was measured using commercial enzyme kits from Abris+ (St. Petersburg, Russia).

The intensity of free radical-induced oxidation processes and total antioxidant activity in the blood serum were determined by biochemiluminescence, induced by hydrogen peroxide and iron(II) sulfate, as described by Piskarev, I.M. (2015) [16]. The biochemiluminescence kinetic curve was recorded for 30 s using a BHL-07 biochemiluminometer (Medozons, Nizhniy Novgorod, Russia). The following biochemiluminescence parameters (BChL) were determined: the chemiluminescence light sum (S), the maximum flash intensity (Imax), and the slope of the tangent to the chemiluminescence curve (tgα2). The reaction medium contained 0.4 mL of 0.02 mM potassium phosphate buffer (pH 7.5), 0.4 mL of 0.01 mM FeSO_4_, and 0.2 mL of a 2% hydrogen peroxide solution. These components were added immediately before measurement. Test material was added to the reaction mixture in a volume of 0.1 mL before the addition of hydrogen peroxide. To analyze the concentration of diene conjugates (DCs), heptane and isopropanol were added to the sample, mixed, and centrifuged at 3000× *g*. The heptane phase was diluted with ethanol and analyzed spectrophotometrically at 233 nm as described by Recknagel R.O. (1966) [17].

Enzyme activity and metabolite levels in serum and tissues were measured using a Shimadzu UV-1900 spectrophotometer (Japan). Catalase activity was measured at 410 nm using a buffer-substrate mixture containing 10 mL Tris-HCl buffer (pH 7.4), 30 mL 0.08% hydrogen peroxide, and 4.5% ammonium molybdate solution as described by Góth L. A (1991) [18]. Aconitate hydratase (AH) activity was assessed in 50 mM Tris-HCl buffer containing 4 mM sodium citrate (PanReac, Barcelona, Spain). Reduced glutathione (GSH) concentration was measured using Ellman’s reagent [19]. Citrate concentration was estimated using the Natelson method [20]. Protein content was determined using the Lowry method [21]. Enzyme activity was expressed as U/mL serum, U/g tissue weight, and U/mg protein, respectively.

### 2.5. Quantitative Real Time PCR

Total RNA from mouse was isolated from the whole blood cells collected in the LPS-induced inflammation model using Extract RNA reagent (Evrogen, Moscow, Russia) according to the manufacturer’s instructions. RNA purity and quantity were assessed using a Nanodrop One spectrophotometer (Thermo Scientific, Waltham, MA, USA). Complementary DNA (cDNA) was synthesized from 1 μg of total RNA pretreated with DNase I (Thermo Scientific, USA) using the MMLV RT kit (Evrogen, Moscow, Russia) according to the manufacturer’s instructions. Quantitative PCR was performed in a CFX96 Touch thermal cycler (Bio-Rad, Hercules, CA, USA) using the Biomaster HS-qPCR SYBR Blue (2×) kit (Biolabmix, Novosibirsk, Russia) and gene-specific primers to the *Tnf* (NM_013693.3), *Il1β* (NM_008361.4), *Il6* (DQ788722.1), *Il10* (NM_010548.2), *Ptgs2* (NM_011198.5),*Nos*2 (NM_001313921.1), and *Actb* (NM_007393.5) genes, synthesized by Evrogen (Moscow, Russia) (Table S1).

PCR was performed in a 20 μL reaction mixture containing 10 μL of Biomaster HS-qPCR SYBR Blue (2×) mix, 2 μL of forward primer (10 μM), 2 μL of reverse primer (10 μM), 5 μL of highly purified water (Ambion, Austin, TX, USA), and 1 μL of cDNA. A negative control without template was used for each primer pair. The reaction was carried out under the following conditions: 95 °C for 5 min, then 40 cycles of denaturation at 95 °C for 10 s, annealing at 52–59 °C for 15 s, and elongation at 72 °C for 25 s, followed by fluorescence reading. Finally, a melting curve was plotted to assess the PCR specificity. Data were analyzed using Bio-Rad CFX96 Manager 3.1 ver. 3.1.1517.0823 (Bio-Rad, Hercules, CA, USA). Relative expression levels of cytokine genes were calculated using the 2^−ΔΔCt^ method by comparing the experimental and control groups [22]. The β-actin gene was used as a reference gene.

### 2.6. Statistical Analysis

Statistical processing of the obtained results was performed using Statistica Version 6.0 (Stat Soft Inc., Tulsa, OK, USA) as well as In-house software developed at the Department of Pharmacology of the Kuban State Medical University. The significance of differences was tested using one-way analysis of variance (ANOVA) with Tukey’s correction. The results of hematological status studies and quantitative PCR were analyzed using one-way analysis of variance (ANOVA), at *p* ≤ 0.05, followed by Dunnett’s multiple comparisons. The results of oxidative status studies were analyzed using SPPS Statistics 23.0 software using the one-sample Kolmogorov–Smirnov test to assess the normality of the distribution of variable values. Values of the parameters in the groups were compared using one-way analysis of variance or the Kruskal–Wallis test. For pairwise comparisons, Student’s *t*-test or the Mann–Whitney test were used. Differences were considered statistically significant at *p* < 0.05.

## 3. Results

### 3.1. Effect of SV-1010 on Acute Exudative Inflammation

**Figure 1:**
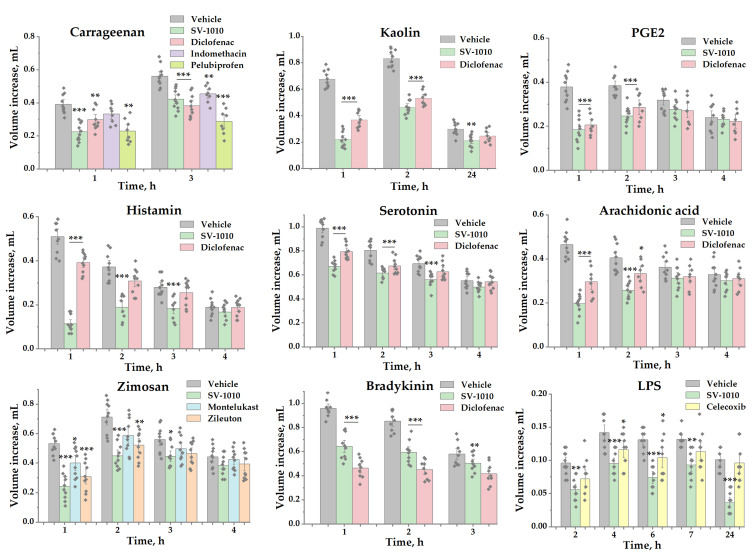
Comparison of the SV-1010 effect at a dose of 0.1 mg/kg with the anti-inflammatory effect of reference drugs selected in the most effective doses in a various inflammation model. Diclofenac and indomethacin were used at a dose of 5 mg/kg (10 mg/kg diclofenac at the bradikinine test), pelubiprofen—at a dose of 3 mg/kg, montelukast—at a dose of 0.22 mg/kg, zileuton—at a dose of 60 mg/kg. SV-1010 and reference drugs were administered intragastrically before 1 h of inflammatory agents administration. The results are presented as the mean ± SEM (*n* = 8–12); *—*p* ≤ 0.05, **—*p* ≤ 0.01, ***—*p* ≤ 0.001 indicates a significant difference in values from the negative control group (vehicle) according to one-way ANOVA/Tukey’s test.

A comparative study of the anti-inflammatory effects of various drugs in models of acute exudative inflammation induced by different inflammatory agents revealed a significant advantage for SV-1010 compared to reference drugs commonly used in clinical practice for inflammation treatment. For comparison, we used the same method of administration of i.g. for all samples at 1 h prior to an inflammatory agent’s administration. Depending on the type of phlogogenes used, the minimal effective dose of SV-1010 varied between 0.001 and 1 mg/kg. However, in all inflammation models, SV-1010 showed stable efficacy at a dose of 0.1 mg/kg, sometimes exceeding the subsequent dose of 1.0 mg/kg. Figure 1 was created to compare the anti-inflammatory effectiveness of an SV-1010 dose of 0.1 mg/kg with that of reference drugs across various tests. Data for other doses of SV-1010 and reference drugs are included in the supplementary materials to this article.

Under conditions of carrageenan-induced edema, SV-1010 exhibited a more pronounced effect one hour after the administration of inflammatory agents compared to the effect after three hours. When comparing the minimum doses of drugs, it was found that the effectiveness of SV-1010 in this test at a dose of 0.1 mg/kg significantly exceeded diclofenac (7.5 mg/kg), indomethacin (10 mg/kg), and pelubiprofen (3 mg/kg), respectively (Table S2).

The anti-inflammatory effect of SV-1010 in the kaolin and prostaglandin E2-induced paw edema model also significantly exceeded that of diclofenac. At doses of 0.1 and 1 mg/kg the effectiveness of SV-1010 was similar to that of the reference drug at a dose of 7.5 mg/kg. However, subsequent observation revealed that the effectiveness of the lower doses of SV-1010 decreased rapidly in the prostaglandin E2-induced edema model. The significant effect was only seen at 0.01 mg/kg after 2 h and no effect was observed after 3 and 4 h. Diclofenac’s efficacy also declined to 7.5 mg/kg after 3 h and did not show a significant difference from the control there after. Furthermore, the anti-inflammatory effects of SV-1010 at doses of 0.1 and 1 mg/kg remained virtually comparable over a 24 h period (Tables S3 and S4).

In rat paw edema induced by histamine, SV-1010 was three orders of magnitude more effective than diclofenac. At the first hour, the minimum effective dose of SV-1010 was 0.001 mg/kg, which was increased to 0.01 mg/kg at two and three hours of observation. Diclofenac also showed a minimal effective dose increase, starting from a dose of 5 mg/kg in the first hour and continuing to 10 mg/kg in the second and third hours (Table S5).

The anti-inflammatory effects of SV-1010 observed in rat paw edema induced by AA or serotonin at a dose of 0.01 mg/kg were similar to those of diclofenac at a dose of 5 mg/kg (Tables S6 and S7). The significant effect of SV-1010 disappeared within 3 h in the case of AA administration, but was more retained in the case of serotonin administration.

In the zymosan-induced edema model, a statistically significant positive effect of SV-1010 was observed at a dose of 0.01 mg/kg in the first hour and at a dose of 0.1 mg/kg in the second hour of observation. The efficacy of the reference drugs montelukast (0.22 mg/kg at the first hour and 0.44 mg/kg at the second hour) and zileuton (60 mg/kg for 2 h) also did not exceed 2 h of observation (Table S8).

Under bradykinin-induced paw edema in rats, a significant anti-inflammatory effect of SV-1010 was observed at doses of 0.1 and 1 mg/kg. The 0.1 mg/kg dose was found to be more effective than the 1 mg/kg dose, and this effect was comparable to that of diclofenac at a dose of 10 mg/kg (Table S9). This indicates that the efficacy of SV-1010 was almost 2 orders of magnitude higher than that of diclofenac in this test.

In the LPS-induced acute exudative inflammation model, SV-1010 showed the effective suppression of paw edema in animals up to 6 h after i.p. administration of the inducer at a dose of 0.01 mg/kg and up to 24 h at a dose of 0.1 mg/kg. Moreover, celecoxib showed reliable inhibition of edema at a dose of 30 mg/kg in the interval up to 4 h after administration of the inducer, with an effect of 30% after 2 h (Table S10).

### 3.2. Effect of SV-1010 on Acute Systemic Inflammation

LPS-induced SI in mice manifested in the form of several clinical symptoms, including decreased motor activity, muscle weakness, impaired coordination, hypothermia, rapid shallow breathing, loose diarrhea, and a reduced response to external stimuli.

In the group of mice receiving diclofenac, the severity of symptoms such as muscle weakness, tachypnea, and reduced motor activity was mild, while symptoms such as impaired coordination and reduced response to stimuli were absent.

The efficacy of the SV-1010 compound in reducing inflammation was lower than that of the reference drug diclofenac. According to a complete blood count, mice with SI showed a decrease in the total number of leukocytes in the peripheral blood compared to healthy animals (Table 1). Additionally, administration of LPS led to a shift in the leukocyte profile towards an increase in the percentages of monocytes, eosinophils, and basophils.

SV-1010 and diclofenac had no effect on leukocyte levels, but they normalized monocyte and eosinophil counts. The percentages of these cells were significantly lower in the treated groups than in the untreated animals. Additionally, no basophils were found in the peripheral blood of animals treated with diclofenac. The basophil count in the SV-1010-treated group did not differ significantly from that in the control group.

Diclofenac treatment caused a shift in leukocyte counts, with an increase in lymphocyte numbers and a decrease in neutrophil numbers. The SV-1010 treatment group showed no significant change in neutrophil or lymphocyte counts.

Furthermore, LPS administration resulted in an increase in platelet count and plateletcrit. SV-1010 reduced thrombocytosis compared to the negative control group, but it was less effective than diclofenac. The hematocrit, red blood cell count, and hemoglobin levels did not differ significantly across all groups.

**Figure 2:**
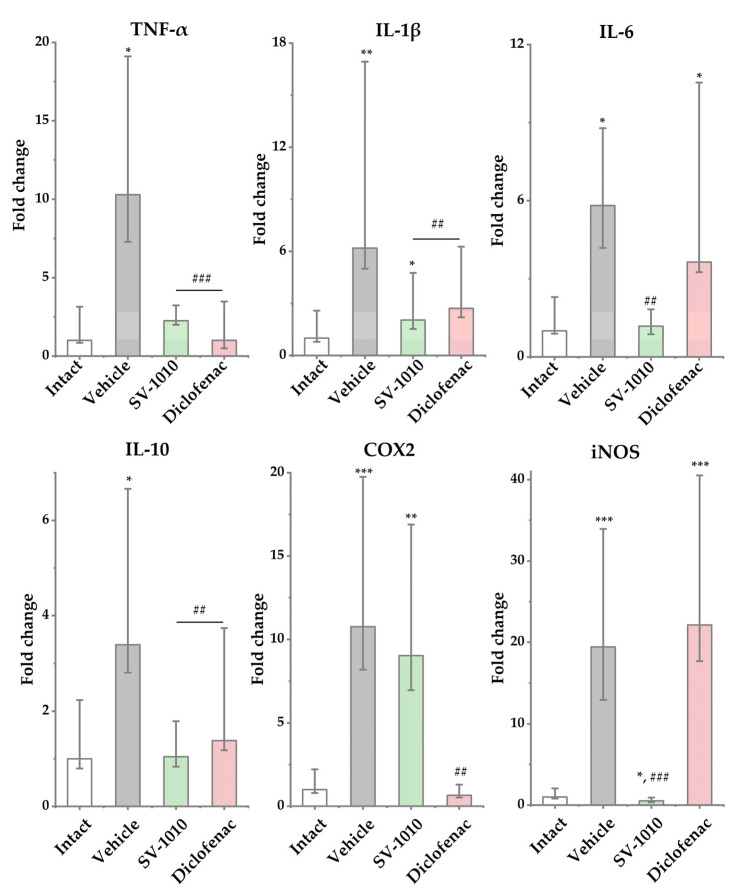
The expression level of genes of some cytokines and pro-inflammatory enzymes normalized as ΔΔCt to the group of healthy animals in the LPC-induced inflammation model. Intact—a group of healthy mice; Vehicle (negative control)—a group of mice treated by water (i.g.); Diclofenac—a group of mice treated by 12.5 mg/kg diclofenac (i.g.); SV-1010—a group of mice treated by 0.1 mg/kg SV-1010 (i.g.). The results are presented as the mean ± SEM (*n* = 10); *—*p* ≤ 0.05, **—*p* ≤ 0.01, ***—*p* ≤ 0.001 indicates a significant difference in values from the intact control; ^##^—*p* ≤ 0.01, ^###^—*p* ≤ 0.001—a significant difference in values from the negative control according to one-way analysis of variance (ANOVA) with subsequent multiple comparisons according to Dunnett.

According to the qPCR results, i.p. administration of LPS significantly increased the expression levels of pro-inflammatory cytokine genes: *Tnf* by 10.3-fold, *Il1β* by 6.2-fold, and *Il6* by 5.8-fold. The anti-inflammatory cytokine, IL-10, also showed a 3.4-fold increase, while the expression of two enzymes, COX-2 and iNOS, increased by 10.8- and 19.4-fold, respectively (Figure 2).

At a dose of 0.1 mg/kg, SV-1010 caused a significant reduction in the expression of *Tnf*, *Il1β*, and *Il6* in experimental animals, comparable to that of diclofenac at a dose of 12.5 mg/kg. However, it did not affect COX-2 gene expression but significantly reduced the expression of iNOS gene (Figure 2). Interestingly, both diclofenac and SV-1010 failed to increase the expression of *Il10*.

Thus, the test compound SV-1010 has an anti-inflammatory effect, normalizing monocyte and eosinophil counts, and suppressing the expression of pro-inflammatory cytokine genes in blood, using a mechanism distinct from that of NSAIDs such as diclofenac.

### 3.3. Effect of SV-1010 on Chronic Proliferative Inflammation

**Figure 3:**
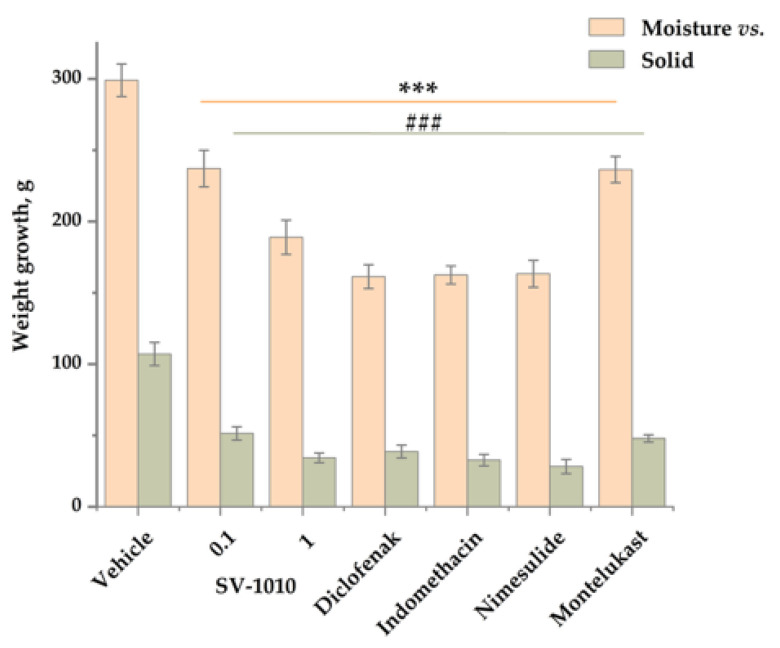
Evaluation of the drugs’ effect on changes in granuloma mass in animals formed on a standard implanted sterile cotton swab weighing 15 mg. SV-1010 (0.1 and 1 mg/kg) and referencedrugs diclofenac (15 mg/kg), indomethacin (20 mg/kg), nimesulide (20 mg/kg) and montelukast (1.76 mg/kg) were administered i.g. 1 h before surgery. The results are presented as the mean ± SEM (*n* = 10); ***—*p* ≤ 0.001 indicates a significant difference in values from the negative control (Moisture); ^###^—*p* ≤ 0.001—a significant difference in values from the negative control (Solid) according to one-way analysis of variance ANOVA with Tukey’s correction.

In the chronic proliferative inflammation model, SV-1010 effectively suppressed both the exudative phase (fluid growth) and the proliferative phase (cell mass growth), similar to the reference drugs. At a dose of 0.1 mg/kg, SV-1010 reduced inflammation by 1.3 times in the exudate phase compared to the control, and at a dose of 1 mg/kg—by 1.6 times. Diclofenac (15 mg/kg), indomethacin (20 mg/kg), nimesulide (20 mg/kg) and montelukast (17.6 mg/kg) also reduced inflammation by 1.9, 1.8, 1.8 and 1.3 times, respectively. Thus, the efficacy of SV-1010 for inhibiting the exudation phase was comparable to that of montelukast at a dose of 0.1 mg/kg, and was 1.3 times higher at a dose of 1 mg/kg. SV-1010 was statistically significantly inferior in efficacy to diclofenac, indomethacin, and nimesulide at a dose of 0.1 mg/kg, while at a dose of 1 mg/kg, the effect was comparable (Figure 3).

### 3.4. The Effect of SV-1010 on Chronic Immune Inflammation

**Figure 4:**
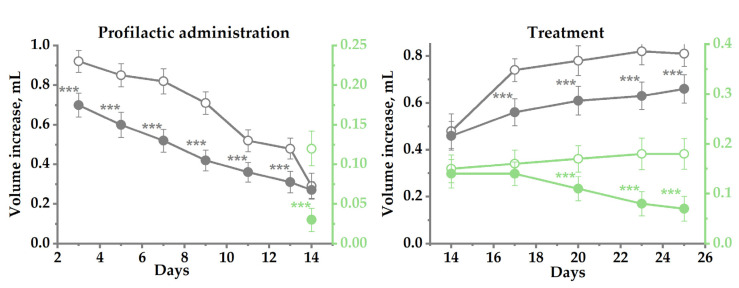
Suppression of the ipsilateral (dark gray) and contralateral (green) paw edema in a rat model of CFA-induced rheumatoid arthritis. The 1% potato starch solution (vehicle) alone (opencircle) or with SV-1010 at a dose of 0.01 mg/kg (color circle) was administrated intragastrically daily for 14 days, beginning 1 day before CFA injection for prophylactic administration. In the treatment regimen, both samples were administered daily during 12 days, beginning 14 days after CFA injection. The results are presented as the mean ± SEM (*n* = 15); ***—*p* ≤ 0.001 indicates a significant difference in values from the negative control according to one-way analysis of variance ANOVA with Tukey’s correction.

The effect of SV-1010 on chronic immune inflammation was studied in a rat model of CFA-induced paw edema, RA type. Prophylactic administration of SV-1010 at a dose of 0.01 mg/kg on 7–9 days after the CFA administration resulted in maximum inhibition of the ipsilateral paw edema (primary immunological response), reaching 30–40% of the control value. Suppression of the contralateral paw edema (secondary immunological response) after administration of SV-1010 on day 14 was 75% (Figure 4). When SV-1010 was administered against the background of rheumatoid arthritis, developed by day 14 in the right paw, and arthritis of immunological origin in the left paw, the inhibition of edema in the right paw did not exceed 24.3% over 25 days, while in the contralateral paw, it reached 72.7%. It should be noted that during the modeling of RA to study the prophylactic and therapeutic effects of SV-1010, no secondary immunological response (edema in the left paw) developed in 5 rats (33.3%) in each group. This is consistent with the literature data [23], and these animals were therefore excluded from the experiment.

After euthanasia, the inflammatory response indices, the levels of cardiomyocyte cytolysis markers, and the oxidative status parameters were measured in the animals of the control group, and groups treated with SV-1010 at a dose of 0.01 mg/kg and with diclofenac at a dose of 12.5 mg/kg (Table 2).

SV-1010, in contrast to diclofenac, significantly reduced ESR, CIC level, and the activity of cytolysis markers CK-MB and AST, which were increased in response to CFA administration. Additionally, SV-1010 normalized all BChL and DCs parameters, whereas diclofenac only reduced Imax.

SV-1010 significantly increased AH activity in the heart and serum, and decreased the serum citrate concentrations in rats with RA. Diclofenac administration to animals with this condition did not result in significant changes in these parameters. SV-1010 also normalized catalase activity, but unlike diclofenac, it did not affect the GSH levels.

Thus, SV-1010 exhibited a corrective effect on most of the analyzed parameters of redox homeostasis in the blood and tissues of animals with RA. Compared to diclofenac, SV-1010 led to more significant changes in most of the analyzed parameters.

## 4. Discussion

Inflammation is a natural process of the immune system, but if it persists for a long time, it can lead to various chronic diseases such as autoimmune disorders, arthritis, cardiovascular and neurodegenerative conditions, diabetes, and cancer [24].

One promising approach to treating inflammation is the use of compounds that suppress the activity of enzymes involved in inflammatory processes or inhibit receptors and ion channels involved in these processes. Initial research has focused on modulating cytokine receptor activity with various agents and antioxidant molecules in order to reduce cellular oxidative stress. However, there has been a lack of attention given to the role of ion channels in peripheral neurons and immune cells in the development of inflammation as a neurogenic process.

The non-selective cation channel, TRPV1 (transient receptor potential vanilloid type 1 channel), has now been identified as one of the main targets of pro-inflammatory mediators [25,26,27,28]. When activated in nociceptive neurons, TRPV1 triggers the release of neuropeptides and neurotransmitters, which in turn generate action potentials and signal pain perception to the central nervous system [8]. Additionally, its activation leads to the peripheral release of pro-inflammatory compounds, which can sensitize other neurons to physical, thermal, or chemical stimuli [29]. TRPV1 is expressed by nociceptors in sensory neurons of the vagus and trigeminal nerves, in the sympathetic plexuses of the intestine and bladder, in neurons of the central nervous system, particularly in the striatum, cerebellum, hippocampus, in dopaminergic neurons of the substantia nigra and hypothalamus, in various layers of the cerebral cortex, as well as in non-neuronal cells such as keratinocytes, immune cells, smooth muscle cells, liver and pancreatic cells, and epithelial cells of different tissues [30,31].

Furthermore, a variety of studies have revealed the expression and function of TRPV1 in various immune cells. TRPV1 expression has been detected in T lymphocytes, macrophages, dendritic cells, natural killer cells, neutrophils, and other immune cells found in both human and mouse immune tissues and blood [32,33].

In the previous study, we showed that SV-1010 as a TRPV1 channel modulator inhibited ionic current through this channel at nanomolar concentrations. In the classic TRPV1 channel assay, using the hot plate test, SV-1010 at a dose of 0.1 mg/kg significantly reduced the animals’ thermosensitivity to high temperatures by 65% compared to the control group. This inhibitory effect was partially attenuated by pretreatment with naloxone, suggesting that the molecule also influences the opioid receptor pathway [10]. TRPV1 has a notable expression in various immune system cells responsible for the development of inflammation, so negative modulation of its activity can lead to changes in the various signaling pathways. Thus, induction of TRPV1 channels triggers the production of pro-inflammatory factors such as TNF-α, IL-6, and NO, which in turn leads to the activation of pro-inflammatory signaling cascades Nf-κB and MAPK [34,35]. TRPV1 activation in endothelial cells has been shown to trigger Ca^2+^-dependent PI3K/Akt/CaMKII signaling, which leads to enhanced phosphorylation of TRPV1, increased TRPV1-eNOS complex formation, eNOS activation and, ultimately, NO production [36]. Inhibition of iNOS gene expression by SV-1010 detected with qPCR in an acute inflammation mice model is likely due to its effect on TRPV1 rather than interaction with a new biological target.

The anti-inflammatory effect of this molecule should be considered as exceeding all expectations. We administered SV-1010 via the gastrointestinal tract, where the molecule was rapidly absorbed and produced a sustained effect for several hours. At low doses of 0.001–0.01 mg/kg, SV-1010 significantly reduced the volume of inflamed paws in tests using kaolin, prostaglandin E2, and histamine; its anti-inflammatory effect had a rapid onset and a sharp decline, while the effect at a higher dose, 0.1 mg/kg was more prolonged. The effect of SV-1010 on the LPS-induced inflammation persisted for 7 and 24 h in contrast to the effect of the comparator drug celoxib, which disappeared by the sixth hour. This may indicate a relatively high clearance of the drug when used, which could be compensated by increasing the dose. Overall, our findings demonstrate the potential of SV-1010 as a novel NSAID type with an alternative mechanism not touching cyclooxygenases.

Based on the sum of all acute inflammation experiments, the average effective dose (ED_50_) of SV-1010, which characterizes the drug’s therapeutic activity in a particular test, was 0.2 mg/kg. Taking into account the estimated LD_50_ of SV-1010 as 1.1 g/kg for rats [10] the resulting therapeutic index is 5500. SV-1010 turned out to be much more effective in terms of therapeutic index over diclofenac, which had a therapeutic index of 70 [10].

The use of various inflammatory agents to induce exudative inflammation has allowed us to characterize SV-1010 as a drug with broad anti-inflammatory activity. It effectively inhibits inflammatory swelling induced by substances such as histamine, serotonin, and bradykinin. These biogenic amines and kinins, as is well-known, trigger inflammatory responses through their own receptors, whose activation lead to a series of biochemical processes that increase the intracellular Ca^2+^ level [29]. A relation between these processes and the activity of the TRPV1 channel has been established [25]. Inhibition of TRPV1 activity by SV-1010 appears to contribute to the suppression of inflammation in these models.

LPS, as a component of the cell wall of Gram-negative bacteria, plays a crucial role in SI by stimulating macrophages via Toll-like receptor 4 (TLR4). This results in a significant increase in reactive oxygen species (ROS) production, pro-inflammatory cytokines (IL-1β and TNF-α) and enzymes involved in AA metabolism (5-lipoxygenase (5-LO) and COX-2) [8,37]. To assess the anti-inflammatory potential of SV-1010, we evaluated its effect on immune system response after LPS administration. This was conducted by analyzing clinical blood parameters and measuring the expression levels of pro-inflammatory cytokine genes in immune cells from the animals’ blood using qPCR.

SV-1010 at a dose of 0.1 mg/kg had a normalizing effect on hematological parameters and effectively reduced the levels of pro-inflammatory cytokines, IL-1β and TNF-α without affecting the COX-2 levels, unlike diclofenac. In an immune inflammatory model similar to adjuvant RA, animals experienced an increase in ESR, CIC level, and activity of cardiomyocyte cytolytic markers CK-MB and AST. Diclofenac treatment resulted in an even greater increase in cardiac markers, while SV-1010 treatment decreased CK-MB, AST and CIC in rats with pathology and reduced ESR to a greater extent than diclofenac.

We believe that SV-1010 does not inhibit COX and eicosanoid synthesis. Instead, the k-opioid receptor may be considered as an additional target for this molecule. In our previous study, we found that subcutaneous administration of naloxone reduced the analgesic effect of SV-1010 by 36.3% (4.05 ± 0.24 for control/8.89 ± 0.51 for SV-1010/7.42 ± 0.33 s for naloxone & SV-1010 in hot plate test) [10]. However, even despite the decrease in analgesia caused by the use of naloxone, the predominant role of TRPV1 inhibition in the observed analgesia can still be considered.

The development of RA in rats was accompanied by an increase in BChL parameters, including the Imax, S, and tgα2. Diclofenac administration led to a decrease in Imax only, while SV-1010 normalized all BChL parameters and reduced the concentration of lipid peroxidation products in rats with RA.

Rats with RA also showed decreased activity of AH, a sensitive target for ROS, and the accumulation of citrate in the tissues. Administration of diclofenac to animals with this RA did not result in significant changes in these parameters. In turn, the administration of SV-1010 increased AH activity in the heart and serum, as well as decreased the serum citrate concentration in rats with RA.

In the modeling of RA pathology, an increase in catalase activity and reduction in GSH in the heart and serum were also observed. This may be a result of the body’s defense mechanisms activating to adapt to the increased formation of free radicals and oxidative stress that occurs during the development of RA. The administration of diclofenac to RA patients led to a decrease in catalase activity, while the GSH level in rat hearts slightly increased compared to those in the RA group. This suggests that there is a need for an increased production of this thiol in order to maintain redox balance in cardiomyocytes. On the other hand, the administration of SV-1010 did not significantly alter catalase activity towards control values, and it had no significant effect on GSH levels.

Pharmacokinetic experiments have not yet been conducted, but based on data from studies using kaolin or LPS-induced inflammation models, where the effects of a single application of SV-1010 at 24 h were still significantly different from those of the control group, we can anticipate an average elimination kinetics for the substance that may even be superior to that of diclofenac, which was used as a control. Therefore, the molecule holds great potential for human treatment. According to the rat-to-human conversion formula, the starting human equivalent dose would be about 0.03 mg/kg. In the meantime, the main focus of our ongoing work is to address the issue of potential toxicity associated with long time molecule administration. It should be noted, however, that the obtained LD_50_ values for SV-1010 at orally administration by male mice (715.0 mg/kg) and by male rats (1109.0 mg/kg) [10] suggests a promising outlook.

## 5. Conclusions

Traditional anti-inflammatory medications, according to their primary cellular target, suppress inflammatory processes by inhibiting prostaglandin’s production. Diclofenac, as a typical NSAID, albeit at a higher dose, successfully addressed all inflammation models in this study. The analyzed antagonist of TRPV1 ion channel, SV-1010, demonstrated greater efficacy at lower doses and a superior therapeutic index compared to diclofenac (5500 vs. 70), maintaining its anti-inflammatory effects for a longer duration. The difference in the molecular targets between the drugs led to different results being observed in the biochemical analysis of blood serum, muscle tissue samples, and the heart, as well as in qPCR assessment of the expression levels of cytokines and enzymes involved in the pro-inflammatory response. We acknowledge the limitations of rodent models and plan to use primary human cells for further validation of this molecule and their practical potential development.

## 6. Patents

Materials regarding the effect of SV-1010 in animal models have been filed for intellectual property protection. Patent application No. RU2025120870 issued 30 July 2025.

## Figures and Tables

**Table 1:** Hematology indices in control and experimental groups of animals.

Parameter	Group			
	Control (Intact)	Control (−)	Diclofenac	SV-1010
WBC, ×10^9^cells/L	6.82 ± 2.72	3.12 ± 2.27 *	3.44 ± 1.29 *	3.31 ± 1.46 *
NEU, %	20.85 ± 7.15	22.86 ± 9.62	14.54 ± 4.90 *	17.28 ± 5.48
LYM, %	71.67 ± 8.65	69.40 ± 7.07	74.77 ± 6.63	73.29 ± 5.55
MON, %	3.23 ± 1.09	5.02 ± 0.69 *	2.77 ± 0.84 ^#^	3.27 ± 0.09 ^#^
EOZ, %	3.71 ± 1.07	6.30 ± 1.30 *	4.84 ± 1.63 *^#^	4.22 ± 2.06 *^#^
BAS,%	0.00 ± 0.00	0.22 ± 0.03	0.00 ± 0.00	0.29 ± 0.07
RBC, 10^12^cells/L	9.67 ± 0.36	9.44 ± 0.32	9.45 ± 0.52	9.71 ± 3.08
HTC, %	0.47 ± 0.01	0.46 ± 0.02	0.47 ± 0.02	0.40 ± 0.15
PLT, ×10^9^ cells/L	481.33 ± 106.31	679.60 ± 215.91 *	361.00 ± 109.02 *^#^	512.57 ± 180.10 ^#^
PCT, %	2.63 ± 0.64	3.77 ± 1.18 *	1.84 ± 0.63 *^#^	2.98 ± 1.27 ^#^
HGB, g/L	167.44 ± 20.59	170.00 ± 21.99	167.11 ± 19.05	170.13 ± 56.18

**Designations.** WBC—white blood cells (total number of white blood cells); Leukocyte formula (percentage ratio of main types of leukocytes): LYM—lymphocytes; NEU—neutrophils; MON—monocytes; EOZ—eosinophils; BAS—basophils. RBC—red blood cells; HTC—hematocrit (the ratio of the volume of formed elements to the unit of total blood volume); PLT—platelets; PCT—plateletcrit (the ratio of the volume of platelets to the unit of total blood volume.); HGB—hemoglobin. **Note.** The results are presented as mean ± SD (*n* = 10), *—indicates a significant difference from the intact control, ^#^—indicates a significant difference from the negative control according to one-way ANOVA with Tukey’s correction, at *p* ≤ 0.05.

**Table 2:** Inflammatory response parameters, levels of cardiomyocyte cytolysis markers, and oxidative status parameters in rats with rheumatoid arthritis treated with diclofenac and SV-1010.

Parameter	Control (Intact)	Control (−)	Diclofenac	SV-1010
ESR, mm/h	3.5 ± 0.87	8.4 ± 2.09 *	3.5 ± 0.81 ^#^	2.33 ± 0.74 ^#^^
CIC, a.u.	19.5 ± 3.9	24.0 ± 5.1 *	22.0 ± 4.9	20.0 ± 5.5 ^#^
CK-MB, U/mL	115.1 ± 22.2	505.4 ± 120.3 *	622.9 ± 122.4 ^#^	207.1 ± 58.3 ^#^^
AST, U/mL	0.245 ± 0.052	0.287 ± 0.074 *	0.405 ± 0.103 ^#^	0.225 ± 0.048 ^#^^
Imax Heart, mV	51.5 ± 11.2	65.4 ± 12.4 *	79.8 ± 10.6 ^#^	50.1 ± 12.4 ^#^^
Imax Serum, mV	68.5 ± 15.8	102.5 ± 23.6 *	93.2 ± 14.2 ^#^	80.8 ± 22.3 ^#^
S Heart, mV*s	485.2 ± 105.1	715.4 ± 157.0 *	800.3 ± 104 ^#^	476.1 ± 107.7 ^#^^
S Serum, mV*s	903.2 ± 205.0	1197.4 ± 247.6 *	1327.4 ± 223.8	948.1 ± 374.5 ^#^^
tgα2 Heart	7.1 ± 1.5	8.5 ± 2.0 *	9.4 ± 1.7	7.1 ± 2.1 ^#^^
tgα2 Serum	14.0 ± 3.1	24.1 ± 5.7 *	26.8 ± 3.2	24.6 ± 6.0
DCs Heart, µmol/g	17.7 ± 4.1	40.5 ± 7.2 *	47.5 ± 5.2 ^#^	20.2 ± 4.2 ^#^^
DCs Serum, µmol/mL	7.5 ± 1.6	14.9 ± 4.3 *	14.1 ± 1.5	7.0 ± 3.1 ^#^^
AH Heart, U/g	2.38 ± 0.53	1.01 ± 0.32 *	0.87 ± 0.61	1.83 ± 0.38 ^#^^
AH Serum, U/mL	1.12 ± 0.25	0.37 ± 0.16 *	0.36 ± 0.23	0.74 ± 0.22 ^#^^
AH Heart, U/mg protein	0.198 ± 0.048	0.125 ± 0.029 *	0.105 ± 0.044	0.194 ± 0.032 ^#^^
AH Serum, U/mg protein	0.060 ± 0.014	0.015 ± 0.010 *	0.014 ± 0.015	0.029 ± 0.012 ^#^^
Citrate Heart, μmol/mL	0.205 ± 0.047	0.318 ± 0.120 *	0.349 ± 0.059	0.296 ± 0.063
Citrate Serum, μmol/mL	0.574 ± 0.137	0.931 ± 0.193 *	0.956 ± 0.178	0.769 ± 0.170 ^#^^
Catalase Heart, U/g	0.652 ± 0.154	0.958 ± 0.207 *	0.877 ± 0.154 ^#^	0.712 ± 0.168 ^#^^
Catalase Serum, U/mL	0.093 ± 0.020	0.227 ± 0.031 *	0.110 ± 0.032 ^#^	0.099 ± 0.023 ^#^
Catalase Heart, U/mg protein	0.074 ± 0.016	0.117 ± 0.028 *	0.102 ± 0.018 ^#^	0.078 ± 0.018 ^#^^
Catalase Serum, U/mg protein	0.008 ± 0.002	0.020 ± 0.005 *	0.012 ± 0.004 ^#^	0.008 ± 0.002 ^#^^
GSH Heart, μmol/mL	0.291 ± 0.065	0.430 ± 0.030 *	0.503 ± 0.069 ^#^	0.400 ± 0.063 ^
GSH Serum, μmol/mL	0.372 ± 0.087	0.474 ± 0.071 *	0.518 ± 0.082	0.523 ± 0.051

Designations. ESR—erythrocyte sedimentation rate; CIC—circulating immune complexes; CK-MB—creatine kinase-MB; AST—aspartate aminotransferase; Imax—maximum flash intensity; S—chemiluminescence light sum; tgα2—tangent to the chemiluminescence curve; DCs—diene conjugates; AH—aconitate hydratase; GSH—reduced glutathione. Note. The results are presented as mean ± SD (*n* = 8), *—*p* < 0.05 indicates a significant difference from the intact control; ^#^—*p* < 0.05 indicates a significant difference from the group with pathology (negative control); ^—*p* < 0.05 indicates a significant difference from the group with pathology treated with diclofenac according to one-way ANOVA with Tukey’s correction.

## Data Availability

No additional supporting data were created during the study.

## Supplementary Materials

The following supporting information can be downloaded at: https://www.mdpi.com/article/10.3390/biomedicines14010060/s1, Table S1: Primer sequences used for the detection of cytokine gene expression; Table S2: Anti-inflammation effects of SV-1010, diclofenac, indomethacin, and pelubiprofen on carrageenan-induced paw edema in rats; Table S3: Anti-inflammation effects of SV-1010 and diclofenac on kaolin-induced paw edema in rats; Table S4: Anti-inflammation effects of SV-1010 and diclofenac on prostaglandin E2-induced paw edema in rats; Table S5: Anti-inflammation effects of SV-1010 and diclofenac on histamine-induced paw edema in rats; Table S6: Anti-inflammation effects of SV-1010 and diclofenac on arachidonic acid-induced paw edema in rats; Table S7: Anti-inflammation effects of SV-1010 and diclofenac on serotonin-induced paw edema in rats; Table S8: Anti-inflammation effects of SV-1010, montelukast, and zileuton on zymosan-induced paw edema in rats; Table S9: Anti-inflammation effects of SV-1010 and diclofenac on bradykinin-induced paw edema in rats; Table S10: Anti-inflammation effects of SV-1010 and celecoxib on LPS-induced paw edema in rats.

## Abbreviations

The following abbreviations are used in this manuscript:

AA	Arachidonic acid
AH	Aconitate hydratase
ANOVA	One-way analysis of variance
AST	Aspartate aminotransferase
BAS	Basophils
BChL	Biochemiluminescence parameters
cDNA	Complementary DNA
CFA	Complete Freund’s adjuvant
CIC	Circulating immune complexes
CK-MB	Creatine kinase-MB
COX-2	Cyclooxygenase-2
DCs	Diene conjugates
ED_50_	Average effective dose
EOZ	Eosinophils
ESR	Erythrocyte sedimentation rate
GSH	Reduced glutathione
HGB	Hemoglobin
HTC	Hematocrit
i.g.	Intragastrically administration
Imax	Maximum flash intensity
iNOS	Inducible nitric oxide synthase
i.p.	Intraperitoneal injection
LD_50_	Average lethal dose
LIM	Lymphocytes
LPS	Lipopolysaccharides
MON	Monocytes
NEU	Neutrophils
NSAIDs	Non-steroidal anti-inflammatory drugs
PCT	Plateletcrit
PGE2	Prostaglandin E2
PLT	Platelets
RA	Rheumatoid arthritis
RBC	Red blood cells
ROS	Reactive oxygen species
S	Chemiluminescence light sum
SI	Systemic inflammation
tgα2	Tangent to the chemiluminescence curve
TI	Therapeutic index
TLR4	Toll-like receptor 4
TNF-α	Tumor Necrosis Factor alpha
TRPV1	Transient receptor potential vanilloid type 1 channel
WBC	White blood cells
