# Supplementary material for: The TRPV1 Channel Modulator Imidazo[1,2-a]Indole Derivative Exhibits Pronounced and Versatile Anti-Inflammatory Activity In Vivo
Source: Biomedicines. 2025 Dec 26;14(1):60. doi: 10.3390/biomedicines14010060 (PMC12839412; doi:10.3390/biomedicines14010060)
Supplement: Supplementary file 1 [file biomedicines-14-00060-s001.zip › biomedicines-3992243-supplementary.pdf]

Supplementary Materials

The TRPV1 Channel Modulator Imidazo[1,2-a]indole Derivative Exhibits Pronounced and Versatile Anti-Inflammatory Activity *in vivo*

Table S1. Primer sequences used for the detection of cytokine gene expression

Gene	Forward (5′–3′)	Reverse (5′–3′)	Annealing temperature, °C	Fragment length, bp
Tnf	GTGGAACTGGCAGAAGA	ACTGATGAGAGGGAGGC	59	192
Il1β	AACCTTTGACCTGGGCTGTC	AAGGTCCACGGGAAAGACAC	56	144
Il6	ATCCAGTTGCCTTCTTGGGA	GGTCTGTTGGGAGTGGTATCC	55	103
Il10	CCCAGGCAGAGAAGCATGG	TCACTCTTCACCTGCTCCACTGC	52	148
Ptgs2	TGAGTACCGCAAACGCTTCT	ACGAGGTTTTTCCACCAGCA	55	148
*Nos*2	ATGTGCTGCCTCTGGTCTTGC	GAACCACTCGTACTTGGGATGC	53	110
Actb	AGGGAAATCGTGCGTGACAT	AACCGCTCGTTGCCAATAGT	52-60	149

Table S2. Anti-inflammation effects of SV-1010, diclofenac, indomethacin, and pelubiprofen on carrageenan-induced paw edema in rats. Paw volume was measured before the injection of phlogogen and at specified intervals. The data are given as the average for the group (n = 8–12) of the individual increase in mL.

**Animal group**	**Dose, mg/kg**	**Average paw volume gain, mL**	
		at 1 h	at 3 h
Vehicle		0.39 ± 0.02<br>(0.35÷ 0.43)*	0.57 ± 0.02<br>(0.52÷ 0.62)
SV-1010	0.01	0.32 ± 0.01<br>(0.29÷ 0.35)<br>[17.9]**	0.50 ± 0.02<br>(0.46÷ 0.54)<br>[12.3]
SV-1010	0.1	0.23 ± 0.02<br>(0.20÷ 0.26)<br>[41]	0.42 ± 0.02<br>(0.38 ÷ 0.46)<br>[26.3]
SV-1010	0.5	0.18 ± 0.02<br>(0.15÷ 0.21)<br>[53.8]	0.37 ± 0.02<br>(0.33 ÷ 0.41)<br>[35.1]
SV-1010	1.0	0.30 ± 0.01<br>(0.27÷ 0.33)<br>[23.1]	0.50 ± 0.02<br>(0.46 ÷ 0.54)<br>[12.2]
Diclofenac	5.0	0.31 ± 0.02<br>(0.26÷ 0.36)<br>[20.5]	0.39 ± 0.02<br>(0.34 ÷ 0.44)<br>[31.5]
Diclofenac	7.5	0.19 ± 0.02<br>(0.15÷ 0.23)<br>[51.3]	0.22 ± 0.02<br>(0.18 ÷ 0.26)<br>[61.4]
Diclofenac	10.0	0.07 ± 0.01<br>(0.04÷ 0.10)<br>[82.1] ¤	0.07 ± 0.01<br>(0.05 ÷ 0.09)<br>[87.7]
Vehicle-2		0.45 ± 0.03<br>(0.38÷ 0.52)	0.63 ± 0.03<br>(0.57÷ 0.69)
Indomethacin	5.0	0.38 ± 0.02<br>(0.32÷ 0.44)<br>[15.6]	0.51 ± 0.02<br>(0.46 ÷ 0.56)<br>[19]
Indomethacin	10.0	0.28 ± 0.02<br>(0.23÷ 0.33)<br>[37.8]	0.36 ± 0.02<br>(0.32 ÷ 0.40)<br>[42.9]
Indomethacin	15.0	0.21 ± 0.02<br>(0.16÷ 0.26)<br>[53.3]	0.23 ± 0.02<br>(0.18 ÷ 0.28)<br>[63.5]
Indomethacin	20.0	0.11 ± 0.02<br>(0.06÷ 0.16)<br>[75.6]	0.11 ± 0.02<br>(0.07 ÷ 0.15)<br>[82.5]
Pelubiprofen	3.0	0.26 ± 0.02<br>(0.21÷ 0.31)<br>[42.2]	0.32 ± 0.03<br>(0.26÷ 0.38)<br>[49.2]

* Confidence intervals at $p < 0.05$ are given in parentheses.

** % inhibitory effect is given in square brackets.

Table S3. Anti-inflammation effects of SV-1010 and diclofenac on kaolin-induced paw edema in rats. Paw volume was measured before the injection of phlogogen and at specified intervals. The data are given as the average for the group (n = 8–10) of the individual increase in mL.

**Animal group**	**Dose, mg/kg**	**Average paw volume gain, mL**		
		at 1 h	at 2 h	at 24 h
Vehicle		0.68 ± 0.02<br>(0.63 ÷ 0.73)*	0.83 ± 0.02<br>(0.79 ÷ 0.87)	0.30 ± 0.02<br>(0.26 ÷ 0.34)
SV-1010	0.001	0.53 ± 0.02<br>(0.49 ÷ 0.57)<br>[22.1]**	0.74 ± 0.02<br>(0.70 ÷ 0.78)<br>[10.8]	0.29 ± 0.02<br>(0.25 ÷ 0.33)<br>[3.3]
SV-1010	0.01	0.33 ± 0.02<br>(0.29 ÷ 0.37)<br>[51.4]	0.61 ± 0.02<br>(0.57 ÷ 0.65)<br>[26.5]	0.29 ± 0.02<br>(0.25 ÷ 0.33)<br>[3.3]
SV-1010	0.1	0.22 ± 0.02<br>(0.18 ÷ 0.26)<br>[67.6]	0.46 ± 0.02<br>(0.42 ÷ 0.50)<br>[44.6]	0.21 ± 0.02<br>(0.17 ÷ 0.25)<br>[30]
SV-1010	1.0	0.24 ± 0.02<br>(0.20 ÷ 0.28)<br>[64.7]	0.49 ± 0.02<br>(0.45 ÷ 0.53)<br>[41]	0.21 ± 0.02<br>(0.16 ÷ 0.26)<br>[29]
Diclofenac	2.5	0.52 ± 0.02<br>(0.47 ÷ 0.57)<br>[23.5]	0.70 ± 0.02<br>(0.66 ÷ 0.74)<br>[15.7]	0.28 ± 0.02<br>(0.24 ÷ 0.32)<br>[6.7]
Diclofenac	5.0	0.37 ± 0.02<br>(0.32 ÷ 0.42)<br>[45.6]	0.55 ± 0.02<br>(0.51 ÷ 0.59)<br>[33.7]	0.25 ± 0.02<br>(0.21 ÷ 0.29)<br>[16.7]
Diclofenac	7.5	0.22 ± 0.02<br>(0.18 ÷ 0.26)<br>[67.6]	0.42 ± 0.02<br>(0.38 ÷ 0.46)<br>[49.6]	0.20 ± 0.02<br>(0.15 ÷ 0.25)<br>[33.3]

* Confidence intervals at $p < 0.05$ are given in parentheses.

** % inhibitory effect is given in square brackets.

Table S4. Anti-inflammation effects of SV-1010 and diclofenac on prostaglandin E2-induced paw edema in rats. Paw volume was measured before the injection of phlogogen and at specified intervals. The data are given as the average for the group (n = 8–10) of the individual increase in mL.

Animal group	Dose, mg/kg	Average paw volume gain, mL at 1 h	at 2 h	at 3 h	at 4 h
Vehicle		0.38 ± 0.02 (0.33 ÷ 0.43)*	0.38 ± 0.02 (0.35 ÷ 0.41)	0.32 ± 0.01 (0.29 ÷ 0.35)	0.24 ± 0.02 (0.19 ÷ 0.29)
SV-1010	0.001	0.28 ± 0.02 (0.24 ÷ 0.32) [26.3]**	0,34 ± 0,02 (0.29 ÷ 0.39) [10.5]	0.33 ± 0.02 (0.29 ÷ 0.37) [-3.1]	0,25 ± 0,02 (0.21 ÷ 0.29) [-4.2]
SV-1010	0.01	0.21 ± 0.02 (0.18 ÷ 0.24) [44.7]	0.28 ± 0.02 (0.24 ÷ 0.32) [26.3]	0.30 ± 0.02 (0.27 ÷ 0.33) [6.3]	0.23 ± 0.02 (0.20 ÷ 0.26) [4.2]
SV-1010	0.1	0.18 ± 0.02 (0.14 ÷ 0.22) [52.6]	0.25 ± 0.02 (0.21 ÷ 0.29) [34.2]	0.28 ± 0.02 (0.24 ÷ 0.32) [12.5]	0.22 ± 0.01 (0.19 ÷ 0.25) [8.3]
SV-1010	1.0	0.20 ± 0.02 (0.17 ÷ 0.23) [47.4]	0.28 ± 0.02 (0.24 ÷ 0.32) [26.3]	0.26 ± 0.02 (0.23 ÷ 0.29) [18.8]	0.23 ± 0.02 (0.20 ÷ 0.26) [4.2]
Diclofenac	2.5	0.28 ± 0.02 (0.24 ÷ 0.32) [26.3]	0.32 ± 0.02 (0.27 ÷ 0.37) [15.7]	0.29 ± 0.02 (0.24 ÷ 0.34) [9.3]	0.23 ± 0.02 (0.19 ÷ 0.27) [4.2]
Diclofenac	5.0	0.21 ± 0.02 (0.17 ÷ 0.25) [44.7]	0.29 ± 0.02 (0.24 ÷ 0.34) [23.6]	0.27 ± 0.02 (0.22 ÷ 0.32) [15.6]	0.22 ± 0.02 (0.17 ÷ 0.27) [8.3]
Diclofenac	7.5	0.16 ± 0.02 (0.12 ÷ 0.20) [57.9]	0.22 ± 0.02 (0.18 ÷ 0.26) [42.1]	0.24 ± 0.02 (0.20 ÷ 0.28) [25.0]	0.21 ± 0.02 (0.17 ÷ 0.25) [12.5]

* Confidence intervals at $p < 0.05$ are given in parentheses.

** % inhibitory effect is given in square brackets.

Table S5. Anti-inflammation effects of SV-1010 and diclofenac on histamine-induced paw edema in rats. Paw volume was measured before the injection of phlogogen and at specified intervals. The data are given as the average for the group (n = 10) of the individual increase in mL.

**Animal group**	**Dose, mg/kg**	**Average paw volume gain, mL**			
		at 1 h	at 2 h	at 3 h	at 4 h
Vehicle		0.51 ± 0.02<br>(0.46 ÷ 0.56)*	0.37 ± 0.02<br>(0.33 ÷ 0.41)	0.28 ± 0.02<br>(0.25 ÷ 0.31)	0.19 ± 0.01<br>(0.16 ÷ 0.22)
SV-1010	0.001	0.32 ± 0.02<br>(0.28 ÷ 0.36)<br>[37.2]**	0.30 ± 0.02<br>(0.26 ÷ 0.34)<br>[18.9]	0.26 ± 0.02<br>(0.23 ÷ 0.29)<br>[7.1]	0.19 ± 0.01<br>(0.17 ÷ 0.21)<br>[0.0]
SV-1010	0.01	0.17 ± 0.02<br>(0.13 ÷ 0.21)<br>[66.7]	0.23 ± 0.01<br>(0.20 ÷ 0.26)<br>[37.8]	0.21 ± 0.01<br>(0.18 ÷ 0.24)<br>[25.0]	0.18 ± 0.01<br>(0.15 ÷ 0.21)<br>[5.3]
SV-1010	0.1	0.11 ± 0.01<br>(0.09 ÷ 0.13)<br>[78.4]	0.19 ± 0.01<br>(0.16 ÷ 0.22)<br>[48,6]	0.18 ± 0.02<br>(0.15 ÷ 0.21)<br>[35.7]	0.17 ± 0.01<br>(0.14 ÷ 0.20)<br>[10.5]
SV-1010	1.0	0.11 ± 0.01<br>(0.09 ÷ 0.13)<br>[78.4]	0.21 ± 0.02<br>(0.17 ÷ 0.25)<br>[43.2]	0.21 ± 0.01<br>(0.18 ÷ 0.24)<br>[25.0]	0.17 ± 0.01<br>(0.14 ÷ 0.20)<br>[10.5]
Diclofenac	5.0	0.39 ± 0.01<br>(0.36 ÷ 0.42)<br>[23.5]	0.31 ± 0.02<br>(0.27 ÷ 0.35)<br>[16.2]	0.26 ± 0.02<br>(0.22 ÷ 0.30)<br>[7.1]	0.19 ± 0.01<br>(0.16 ÷ 0.22)<br>[0.0]
Diclofenac	10.0	0.19 ± 0.01<br>(0.16 ÷ 0.22)<br>[62.7]	0.22 ± 0.01<br>(0.20 ÷ 0.24)<br>[40.5]	0.20 ± 0.01<br>(0.18 ÷ 0.22)<br>[28.5]	0.18 ± 0.01<br>(0.16 ÷ 0.20)<br>[5.2]
Diclofenac	15.0	0.13 ± 0.01<br>(0.10 ÷ 0.16)<br>[74.5]	0.22 ± 0.01<br>(0.19 ÷ 0,25)<br>[40.5]	0.22 ± 0.01<br>(0.19 ÷ 0.25)<br>[21.4]	0.17 ± 0.01<br>(0.15 ÷ 0.19)<br>[5.2]

* Confidence intervals at $p < 0.05$ are given in parentheses.

** % inhibitory effect is given in square brackets.

Table S6. Anti-inflammation effects of SV-1010 and diclofenac on arachidonic acid-induced paw edema in rats. Paw volume was measured before the injection of phlogogen and at specified intervals. The data are given as the average for the group (n = 8-10) of the individual increase in mL.

Animal group	Dose, mg/kg	Average paw volume gain, mL at 1 h	at 2 h	at 3 h	at 4 h
Vehicle		0.47 ± 0.02	0.40 ± 0.02	0.36 ± 0.02	0.33 ± 0.02
		(0.42 ÷ 0.52)*	(0.36 ÷ 0.44)	(0.32 ÷ 0.40)	(0.29 ÷ 0.37)
SV-1010	0.001	0.40 ± 0.01	0.43 ± 0.01		
		(0.38 ÷ 0.42)	(0.41 ÷ 0.45)		
		[14.9]**	[-7.5]		
SV-1010	0.01	0.27 ± 0.02	0.32 ± 0.02	0.34 ± 0.02	0.32 ± 0.02
		(0.22 ÷ 0.32)	(0.28 ÷ 0.36)	(0.30 ÷ 0.38)	(0.29 ÷ 0.35)
		[42.5]	[20.0]	[5.6]	[3.0]
SV-1010	0.1	0.20 ± 0.02	0.26 ± 0.01	0.31 ± 0.01	0.30 ± 0.01
		(0.16 ÷ 0.24)	(0.23 ÷ 0.29)	(0.28 ÷ 0.34)	(0.27 ÷ 0.33)
		[57.4]	[35.0]	[13.9]	[9.1]
SV-1010	1.0	0.21 ± 0.02	0.27 ± 0.02	0.33 ± 0.01	0.31 ± 0.01
		(0.17 ÷ 0.25)	(0.23 ÷ 0.31)	(0.31 ÷ 0.35)	(0.28 ÷ 0.34)
		[55.3]	[32.5]	[8.3]	[6.1]
Diclofenac	5.0	0.30 ± 0.02	0.33 ± 0.02	0.32 ± 0.02	0.31 ± 0.02
		(0.24 ÷ 0.36)	(0.28 ÷ 0.38)	(0.27 ÷ 0.37)	(0.26 ÷ 0.36)
		[36.1]	[17.5]	[11.1]	[6.1]
Diclofenac	10.0	0.23 ± 0.02	0.26 ± 0.02	0.28 ± 0.02	0.30 ± 0.02
		(0.18 ÷ 0.28)	(0.21 ÷ 0.31)	(0.23 ÷ 0.33)	(0.25 ÷ 0.35)
		[51.1]	[35.0]	[22.2]	[9.1]
Diclofenac	15.0	0.15 ± 0.02	0.23 ± 0.02	0.26 ± 0.02	0.28 ± 0.02
		(0.10 ÷ 0.20)	(0.17 ÷ 0.29)	(0.21 ÷ 0.31)	(0.24 ÷ 0.32)
		[68.1]	[42.5]	[27.8]	[15.2]

* Confidence intervals at $p < 0.05$ are given in parentheses.

** % inhibitory effect is given in square brackets.

Table S7. Anti-inflammation effects of SV-1010 and diclofenac on serotonin-induced paw edema in rats. Paw volume was measured before the injection of phlogogen and at specified intervals. The data are given as the average for the group (n = 10) of the individual increase in mL.

**Animal group**	**Dose, mg/kg**	**Average paw volume gain, mL** at 1 h	at 2 h	at 3 h	at 4 h
Vehicle		0.99 ± 0.02 (0.94 ÷ 1.04)*	0.81 ± 0.02 (0.76 ÷ 0.86)	0.70 ± 0.02 (0.65 ÷ 0.75)	0.55 ± 0.02 (0.50 ÷ 0.60)
SV-1010	0.01	0.78 ± 0.02 (0.73 ÷ 0.83) [21.2]**	0.69 ± 0.02 (0.65 ÷ 0.73) [14.8]	0.62 ± 0.02 (0.58 ÷ 0.66) [11.4]	0.51 ± 0.02 (0.47 ÷ 0.55) [7.3]
SV-1010	0.1	0.67 ± 0.02 (0.63 ÷ 0.71) [32.3]	0.62 ± 0.01 (0.59 ÷ 0.65) [23.4]	0.57 ± 0.01 (0.54 ÷ 0.61) [18.6]	0.50 ± 0.02 (0.46 ÷ 0.54) [9.1]
SV-1010	0.5	0.45 ± 0.02 (0.41 ÷ 0.49) [54.5]	0.50 ± 0.02 (0.46 ÷ 0.54) [38.3]	0.54 ± 0.02 (0.49 ÷ 0.59) [22.8]	0.52 ± 0.02 (0.47 ÷ 0.57) [5.4]
SV-1010	1.0	0.71 ± 0.02 (0.66 ÷ 0.76) [28.3]	0.66 ± 0.01 (0.63 ÷ 0.69) [18.5]	0.64 ± 0.01 (0.61 ÷ 0.67) [8.6]	0.53 ± 0.02 (0.51 ÷ 0.57) [3.6]
Diclofenac	5.0	0.79 ± 0.02 (0.75 ÷ 0.83) [20.2]	0.68 ± 0.02 (0.64 ÷ 0.72) [16.0]	0.63 ± 0.02 (0.58 ÷ 0.68) [10.0]	0.54 ± 0.02 (0.49 ÷ 0.59) [1.8]
Diclofenac	10.0	0.44 ± 0.01 (0.41 ÷ 0.47) [55.5]	0.52 ± 0.02 (0.48 ÷ 0.56) [35.8]	0.58 ± 0.01 (0.55 ÷ 0.61) [17.1]	0.52 ± .02 (0.48 ÷ 0.56) [5.4]
Diclofenac	15.0	0.30 ± 0.02 (0.26 ÷ 0.34) [69.7]	0.40 ± 0.02 (0.36 ÷ 0.44) [50.6]	0.53 ± 0.02 (0.49 ÷ 0.57) [24.3]	0.50 ± 0.02 (0.46 ÷ 0.54) [10.0]

* Confidence intervals at $p < 0.05$ are given in parentheses.

** % inhibitory effect is given in square brackets.

Table S8. Anti-inflammation effects of SV-1010, montelukast, and zileuton on zymosan-induced paw edema in rats. Paw volume was measured before the injection of phlogogen and at specified intervals. The data are given as the average for the group (n = 10) of the individual increase in mL.

Animal group	Dose, mg/kg	Average paw volume gain, mL			
		at 1 h	at 2 h	at 3 h	at 4 h
Vehicle		0.53 ± 0.02<br>(0.48 ÷ 0.58)*	0.71 ± 0.03<br>(0.63 ÷ 0.79)	0.56 ± 0.03<br>(0.50 ÷ 0.62)	0.44 ± 0.02<br>(0.38 ÷ 0.50)
SV-1010	0.001	0.46 ± 0.03<br>(0.40 ÷ 0.52)<br>[13.2]**	0.67 ± 0.03<br>(0.59 ÷ 0.75)<br>[5.6]		
SV-1010	0.01	0.36 ± 0.03<br>(0.30 ÷ 0.42)<br>[32.1]	0.59 ± 0.02<br>(0.54 ÷ 0.64)<br>[16.9]	0.48 ± 0.03<br>(0.41 ÷ 0.55)<br>[16.7]	0.44 ± 0.04<br>(0.36 ÷ 0.52)<br>[0.0]
SV-1010	0.1	0.24 ± 0.03<br>(0.19 ÷ 0.31)<br>[54.7]	0.45 ± 0.03<br>(0.39 ÷ 0.51)<br>[36.6]	0.45 ± 0.02<br>(0.40 ÷ 0.50)<br>[19.6]	0.38 ± 0.02<br>(0.33 ÷ 0.43)<br>[13.6]
Montelukast	0.22	0.40 ± 0.03<br>(0.33 ÷ 0.47)<br>[24.5]	0.59± 0.04<br>(0.51 ÷ 0.67)<br>[16.9]	0.50 ± 0.03<br>(0.44 ÷ 0.56)<br>[10.7]	0.43± 0.02<br>(0.38 ÷ 0.48)<br>[2.3]
Montelukast	0.44	0.22 ± 0.03<br>(0.17 ÷ 0.31)<br>[58.5]	0.46 ± 0.03<br>(0.39 ÷ 0.53)<br>[35.2]	0.45 ± 0.03<br>(0.39 ÷ 0.51)<br>[19.6]	0.41 ± 0.02<br>(0.36 ÷ 0.46)<br>[6.8]
Montelukast	0.88	0.19 ± 0.03<br>(0.12 ÷ 0.28)<br>[64.2	0.42 ± 0.03<br>(0.36 ÷ 0.48)<br>[40.8]	0,45 ± 0.02<br>(0,40 ÷ 0.50)<br>[19,6]	0.40 ± 0.02<br>(0.35 ÷ 0.45)<br>[9.1]
Zileuton	60.0	0.31 ± 0.03<br>(0.25 ÷ 0.39)<br>[41.5]	0.52 ± 0.03<br>(0.45 ÷ 0.59)<br>[26.8]	0.46 ± 0.02<br>(0.41 ÷ 0.51)<br>[17.9]	0.39 ± 0.02<br>(0.34 ÷ 0.44)<br>[11.4]

* Confidence intervals at $p < 0.05$ are given in parentheses.

** % inhibitory effect is given in square brackets.

Table S9. Anti-inflammation effects of SV-1010 and diclofenac on bradykinin-induced paw edema in rats. Paw volume was measured before the injection of phlogogen and at specified intervals. The data are given as the average for the group (n = 9-10) of the individual increase in mL.

Animal group	Dose, mg/kg	Average paw volume gain, mL		
		at 1 h	at 2 h	at 3 h
Vehicle		0.96 ± 0.03 (0.89 ÷ 1.03)*	0.85 ± 0.03 (0.79 ÷ 0.91)	0.58 ± 0.03 (0.51 ÷ 0.65)
SV-1010	0.1	0.63 ± 0.03 (0.57 ÷ 0.71) [34.4]**	0.60 ± 0.03 (0.53 ÷ 0.67) [29.4]	0.50 ± 0.02 (0.44 ÷ 0.56) [13.8]
SV-1010	1.0	0.69 ± 0.03 (0.62 ÷ 0.76) [28.1]	0.71 ± 0.02 (0.65 ÷ 0.77) [16.5]	0.53 ± 0.02 (0.47 ÷ 0.59) [8.6]
Diclofenac	10.0	0.46 ± 0.03 (0.39 ÷ 0.53) [52.1]	0.45± 0.02 (0.40 ÷ 0.50) [47.1]	0.42 ± 0.03 (0.35 ÷ 0.49) [27.6]

* Confidence intervals at $p < 0.05$ are given in parentheses.

** % inhibitory effect is given in square brackets.

Table S10. Anti-inflammation effects of SV-1010 and celecoxib on LPS-induced paw edema in rats. Paw volume was measured before the injection of phlogogen and at specified intervals. The data are given as the average for the group (n = 10) of the individual increase in mL.

Animal group	Dose, mg/kg	Average paw volume gain, mL				
		at 2 h	at 4 h	at 6 h	at 7 h	at 24 h
Vehicle		0.10 ± 0.005<br>(0.09 ÷ 0.11)*	0.14 ± 0.006<br>(0.13 ÷ 0.15)	0.13 ± 0.005<br>(0.12 ÷ 0.14)	0.13 ± 0.004<br>(0.12 ÷ 0.14)	0.10 ± 0.004<br>(0.09 ÷ 0.11)
SV-1010	0.01	0.05 ± 0.005<br>(0.04÷ 0.06)<br>[50.0]**	0.09 ± 0.006<br>(0.08 ÷ 0.10)<br>[35.7]	0.08 ± 0.005<br>(0.07 ÷ 0.09)<br>[38.5]	0.11 ± 0.005<br>(0.10 ÷ 0.12)<br>[15.4]	0.09 ± 0.005<br>(0.08 ÷ 0.10)<br>[10.0]
SV-1010	0.1	0.06 ± 0.005<br>(0.05 ÷ 0.07)<br>[40.0]	0.09 ± 0.005<br>(0.08 ÷ 0.10)<br>[35.7]	0.07 ± 0.005<br>(0.06 ÷ 0.08)<br>[46.2]	0.09 ± 0.006<br>(0.08 ÷ 0.10)<br>[30.8]	0.04 ± 0.004<br>(0.03 ÷ 0.05)<br>[60.0]
Celecoxib	30.0	0.07 ± 0.006<br>(0.06 ÷ 0.08)<br>[30.0]	0.11 ± 0.006<br>(0.10 ÷ 0.12)<br>[21.4]	0.10 ± 0.008<br>(0.08 ÷ 0.12)<br>[23.1]	0.11 ± 0.008<br>(0.09 ÷ 0.13)<br>[15.4]	0.09 ± 0.006<br>(0.08 ÷ 0.10)<br>[10.0]

* Confidence intervals at $p < 0.05$ are given in parentheses.

** % inhibitory effect is given in square brackets.
